# Development and implementation of a cancer pain intervention registry: An Australian pilot study with preliminary outcome evaluation from 2 tertiary centers

**DOI:** 10.1017/S1478951526101631

**Published:** 2026-02-05

**Authors:** Yi-Ching Lee, Emma Zhao, Timothy Brake, Alix Dumitrescu, Wei Lee, Paul Glare, Robert Sanders, Andy Yi-Yang Wang

**Affiliations:** 1 Department of Anaesthetics and Pain Management Centre, Royal Prince Alfred Hospital, Sydney, Australia; 2 Department of Anaesthetics and Pain Service, Chris O’Brien Lifehouse, Sydney, Australia; 3 School of Medicine, Faculty of Medicine & Health, University of Sydney, Sydney, Australia; 4 Improving Palliative, Aged and Chronic Care through Clinical Research and Translation (IMPACCT), Faculty of Health, University of Technology Sydney, Sydney, Australia; 5 Sydney Nursing School, Faculty of Medicine & Health, University of Sydney, Sydney, Australia; 6 Department Palliative Care, Royal Prince Alfred Hospital, Sydney, Australia; 7 HammondCare, Royal North Shore Hospital, Sydney, Australia; 8 Department of Anaesthesia, Pain and Perioperative Medicine, Royal North Shore Hospital, Sydney, Australia

**Keywords:** Cancer pain, intervention, procedure database, registry, Australia

## Abstract

**Objectives:**

To pilot a registry to evaluate the use and effectiveness of interventional cancer pain management.

**Methods:**

Upon interventional pain procedure scheduling, patient demographics, cancer, and pain information were entered into the longitudinal clinical registry in 2 tertiary hospitals in Sydney, Australia (Royal Prince Alfred Hospital and Chris O’Brien Lifehouse). Details of the procedure (including proceduralist, nature of the intervention, and site of treatment), post-procedure patient-reported outcomes and quality of life surveys, adverse events, and mortality data (when known) were collected longitudinally.

**Results:**

Between October 2021 and March 2023, 48 patients underwent 55 procedures. Procedures included treatment targeting autonomic plexuses, peripheral nerves, fascial planes, and neuraxial structures. Celiac plexus neurolysis was the most frequently reported procedure (33.3%). Post-procedure, there was a trend in reduction in pain intensity on the Patient-Reported Outcome Measurement Information System (*p* < 0.01), reduction in opioid consumption, and improvement in quality of life on the European Organization for Research and Treatment of Cancer Quality of Life Questionnaire-Core-15-Palliative Care.

**Significance of results:**

This is a vital first step in creating a more widely applicable registry evaluating cancer pain intervention. It provided valuable information on the range of available pain intervention procedures and data on patient-reported outcome measures using validated instruments. This will facilitate a timely review of clinical practice to improve future patient care. An Australian-wide database of cancer pain will be a valuable next step in the improvement of cancer pain management.

**Trial registration:**

Clinical trial number: not applicable.

## Introduction

In Australia, approximately 440 people per day are diagnosed with cancer, and more than 1 million people are living with a diagnosis of cancer (cancer survivors) (Australian Institute of Health and Welfare 2022). Globally, by 2040, an estimated 26 billion people will be cancer survivors (Shapiro 2018), with up to 50% experiencing pain (Haumann et al. 2017; Shapiro 2018). The etiology of pain in cancer patients is diverse, relating to the disease, its treatment, or an unrelated comorbidity (Glare et al. 2014; Van Den Beuken-Van Everdingen et al. 2016). Pain may have a nociceptive mechanism (e.g., due to ongoing tissue damage), or may have neuropathic or mixed contributions, sustained by aberrant somatosensory processing in the peripheral or central nervous systems (Falk et al. 2014; Rasmussen et al. 2023).

While opioids have been established as the cornerstone of pain management for patients with moderate to severe cancer pain (Organization 2018), they have limited efficacy and there are concerns of opioid-induced hyperalgesia (Yi and Pryzbylkowski 2015; Higgins et al. 2019), other opioid-related adverse effects (Glare et al. 2014), and potential roles in tumor progression (Singleton et al. 2015). As the prognosis of cancer improves, patients are living longer, raising concerns about prolonged use (tolerance, addiction, accidental overdose) as in nonmalignant pain populations (Farquhar-Smith and Brown 2017). Therefore, there is an increasing shift toward a multimodal approach to managing cancer pain, including utilization of interventional pain procedures (Glare et al. 2014; Lee et al. 2021).

Despite the potential benefits of these interventional procedures, clinician practice, including the timing of referral and referral pathways, appears to vary significantly. Furthermore, outcomes from such procedures are not routinely collected and published. While oncology supportive care services serve as an emerging model to address the issue of fragmented symptom care (Hui et al. 2021), little attention has been paid to interventional pain services within the model. As such, we have established a pilot cancer pain intervention database at 2 tertiary centers in Sydney, Australia. Our vision is that data from this registry will guide and assist decision making in the clinical pathway for the treatment of cancer pain.

The purpose of this study was to use this pilot cancer pain intervention database to assess the feasibility of developing a registry for cancer pain intervention. Such a database will allow timely evaluation of clinical outcomes and practice at a local level, thus helping to improve future patient care.

## Methods

This was a pilot, prospective, longitudinal clinical registry of patients undergoing interventional procedures to manage cancer pain at 2 centers in Sydney (Chris O’Brien Lifehouse and Royal Prince Alfred Hospital). The study was conducted between October 2021 and March 2023. The study conformed to the principles outlined in the Declaration of Helsinki and was approved by the Sydney Local Health District Human Research Ethics Committee, Australia (2021/ETH11313).

### Registry development

The pilot registry, including variables to be included, was developed by a pain specialist (YCL) and an anesthetist/biostatistician (AW) in conjunction with cancer care providers working in palliative care and pain medicine at Chris O’Brien Lifehouse and Royal Prince Alfred Hospital. The assessment instruments were chosen for their relevance in cancer populations, ease and practicality of use, and validity in this setting. Registry data were collected and managed using Research Electronic Data Capture (REDCap) Software hosted at Sydney Local Health District (Harris et al. 2009, 2019). REDCap is a secure, web-based software platform designed by Vanderbilt University to support data capture for clinical research. It provides an intuitive interface for validated data capture; audit trails that allow tracking of data manipulation and export procedures; automated export procedures to facilitate seamless data downloads to common statistical packages; and procedures for importing data from external sources. Participants either completed a paper form or were emailed a link that allowed them to enter the data directly into REDCap. Information on the paper forms was entered into REDCap by the study staff.

### Participants

All patients undergoing interventional procedures for the management of pain associated with cancer or cancer treatment at the 2 participating centers were included in the registry under an “opt-out” consent process. Due to the nature of the registry, an opt-out approach to consent was taken to maximize data capture, minimize biased sampling, while still ensuring each participant had the time to consider if they did not wish to have their data collected. All potential participants were provided with written information about the registry during a routine clinic visit by one of their treating clinicians.

### Registry data collection (variables)

Data collection for an individual patient commenced at the time of scheduling their interventional procedure. At baseline, defined as anytime before the intervention (usually within 2 weeks), information was collected on the patient’s demographics, cancer-related characteristics (type, stage, duration, treatment, recurrence, and Australia-modified Karnofsky Performance Status [AKPS]) (Abernethy et al. 2005); pain-related attributes (duration, type of pain, severity, distress, disability, and psychological mediators of distress and disability); medication use; symptom burden (using the *Patient-Reported Outcome Measurement Information System – Pain Intensity and Pain Interference* questionnaire [*PROMIS-PI*]; Amtmann et al. 2010; Khanna et al. 2011; Habibi et al. 2023); and quality of life (using the *European Organization for Research and Treatment of Cancer Quality of Life Questionnire-Core-15-Palliative Care* [*EORTC QLQ-C15-PAL*]; Fayers et al. 2001; Karen et al. 2006).

The AKPS is an amalgam of the original Karnofsky Performance Status (KPS) and the Thorne-modified KPS, which is commonly used in inpatient and community palliative care settings to assist with clinical decision making (Abernethy et al. 2005). The AKPS is a categorical scale with 11 levels, which range from 100 – normal; no complaints; no evidence of disease to 0 – dead (Abernethy et al. 2005).

*PROMIS-PI* measures the self-reported consequences of pain on a person’s life, including pain intensity and pain interference.

*EORTC QLQ-C15-PAL* is a 15-item survey with 4-item Likert responses, from 1 (not at all) to 4 (very much) (Groenvold et al. 2006). The last question asks about quality of life and is scored on a 7-item Likert scale from 1 (very poor) to 7 (excellent) (Groenvold et al. 2006).

Information on the interventional pain management procedure; any complications; medication use; symptom burden; self-assessment of treatment (using the self-assessment of treatment [SAT] tool; Wyrwich et al. 2012); and quality of life was collected at various time-points after the procedure, including one immediately post-procedure (within 2 weeks) and one after 3 months if appropriate. The SAT is a 5-item simple question scale using a 5-point Likert scale, where the lower option (−2) indicated a negative response, the middle option (0) indicated a neutral response and the higher option (+2) positive response (Wyrwich et al. 2012). Efforts were made to align the timing of the data collection with patients’ routine clinical follow-up.

### Statistical methods

The data were summarized descriptively. No imputation was used for missing data. PROMIS scores were converted to T-scores as per its instrument scoring manual, and EORTC QLQ-C15-PAL scores were transformed to a 0 to 100 scale as per its scoring procedure. Changes from baseline in outcomes of interest were tested univariately by regressing against the time when the measurement occurred (baseline or 1 week), and then using a multivariate linear mixed effect model adjusted for age, sex, and baseline AKPS score, and accounted for within-procedure variations. For both univariate and multivariate models, information was included at the procedure (not patient) level. *p*-Values of <0.05 were considered statistically significant. Analyses were conducted using R Statistical Software (version 4.2.2; R Core Team 2021).

## Results

### Patients

Between October 2021 and March 2023, 48 patients were entered into the registry, with information on 55 procedures being captured, as some patients had multiple procedures. Participant demographics and baseline clinical characteristics are reported in Table 1. Included patients typically had good performance status (approximately 50% had AKPS scores of 70 or better), and had been experiencing pain, typically cancer-related pain, for more than 8 months. The most common site of pain was the abdomen.

**Table 1.** Patient demographics and baseline clinical characteristics

Patient demographics (*n* = 48)	
**Age (years)**, mean ± standard deviation [SD]	61 ± 15
**Female sex**, *n* (%)	25 (52.1)
**Body mass index (kg/m^2^**), mean ± SD	25.7 ± 5.8
**AKPS**	
0 (dead)	0 (0)
10 (comatose or barely rousable)	0 (0)
20 (totally bedfast and requiring extensive nursing)	0 (0)
30 (almost completely bedfast)	2 (4.2)
40 (in bed >50% of time)	3 (6.3)
50 (considerable assistance and frequent medical care required)	7 (14.6)
60 (able to care for most needs)	11 (22.9)
70 (cares for self)	8 (16.7)
80 (normal activity with effort)	16 (33.3)
90 (able to carry on normal activity)	1 (2.1)
100 (normal)	0 (0)
Cancer information (*n* = 53)*	
**Age at cancer diagnosis (years**), mean ± SD	57 ± 15
**Number of cancer diagnoses**	1.19 ± 0.4
**Current cancer type^a^**	
Primary	22 (45)
Metastasis	21 (43)
Recurrence	5 (10)
Uncertain	1 (2)
Unknown	4
**Concurrent oncological treatment**, *n* (%)	25 (47.2)
Pain information (*n* = 53)*	
**Pain duration (months)**, median (IQR)	5.5 (3.0–10.0)
**Origin of pain^a^**, *n* (%)	
Cancer-related	32 (60)
Treatment-related	9 (17)
Unknown	12 (23)
**Sites of pain^b^**, *n* (%)	
Abdomen	25 (52)
Back, thoracic or lumbar region	16 (33)
Pelvic and genital region	11 (23)
Lower limb(s)	8 (17)
Chest	8 (17)
Head and neck	5 (19)
Upper limb(s)	2 (4)
**Sides of pain^a^**, *n* (%)	
Left	18 (34)
Axial	16 (30)
Right	10 (19)
Bilateral	6 (11)
Indeterminate	3 (6)
**Cancer pain type^a^**, *n* (%)	
Neuropathic^c^	18 (34)
Visceral^d^	16 (30)
Bone	10 (19)
Post chemotherapy	4 (7.5)
Post radiotherapy	3 (5.7)
Post-surgical	1 (1.9)
Unknown	1 (1.9)

AKPS = Australia-modified Karnofsky Performance Status.

a Total number here is lower than the total procedures performed (*n* = 55) due to missing data.

b Does not sum to 100% as patients could have more than one pain location.

c Thoracic tumor with brachial plexus invasion or pelvic cancer affecting lumbosacral plexus or spinal cord compression.

d Liver metastasis, pancreatic tumor.

* Patients could have more than one cancer type.

### Procedures

The registry collected information on 55 procedures (Table 2). Most procedures were performed in an operating theater (50.9%) by pain specialists (75.5%: Table 2). Most procedures required imaging guidance, typically by computed tomography (CT – 46.2%) or ultrasound (25%). The most common procedure performed was celiac plexus neurolysis (33.3%).

**Table 2.** Cancer pain intervention procedures (*n* = 55)^a^

Procedure location	*n* (%)
Operating theater	27 (50.9)
Radiology department	23 (43.4)
Recovery/anesthetic bay	3 (5.7)
**Proceduralist^b^**	
Pain specialist	40 (75.5)
Neurosurgeon	6 (11.3)
Radiologist	3 (5.7)
Gastroenterologist	2 (3.8)
Anesthetist	1 (1.9)
**Imaging modality^c^**	
CT	26 (46.2)
X-ray/fluoroscopy	11 (19.6)
Ultrasound	14 (25)
Nil	4 (7.1)
Angiogram	1 (1.8)
**Target sites (*n* = 57)^d^**	
**Autonomic nerve/plexus**	**21 (36.8)**
Celiac plexus	19 (90.4)
Splanchnic plexus blocks	1 (4.8)
Superior hypogastric plexus	1 (4.8)
**Peripheral nerve**	**21 (36.8)**
Nerve root	7 (33.3)
Intercostal	4 (19)
Trigeminal^e^	4 (19)
Suprascapular	2 (9.5)
Femoral	1 (4.8)
Pudendal	1 (4.8)
Other (ilioinguinal and perivascular)	2 (9.5)
**Neuraxial**	**11 (19.3)**
Cordotomy	6 (54.5)
*Percutaneous cervical*	*4*
*Open thoracic cordotomy*	*2*
Intrathecal intervention	5 (45.5)^f^
**Fascial plane**	**4 (7)**
Serratus anterior	1 (25)
Erector spinae	1 (25)
Fascia iliaca	2 (50)
**Other**	**1 (1.8)**

a Two patients on the registry did not receive intervention.

b *n* > 55 as some procedures involved more than one proceduralist.

c *n* = 56 as one procedure involved both fluoroscopy and ultrasound.

d *n* = 57 as some procedures included more than 1 target site.

e Trigeminal nerves include mental, mandibular, supratrochlear and supraorbital nerves.

f Four intrathecal neurolysis (saddle block) and 1 intrathecal catheter placement (as a temporalizing measure to bridge and facilitate cordotomy).

### Outcomes

**Figure 1.**
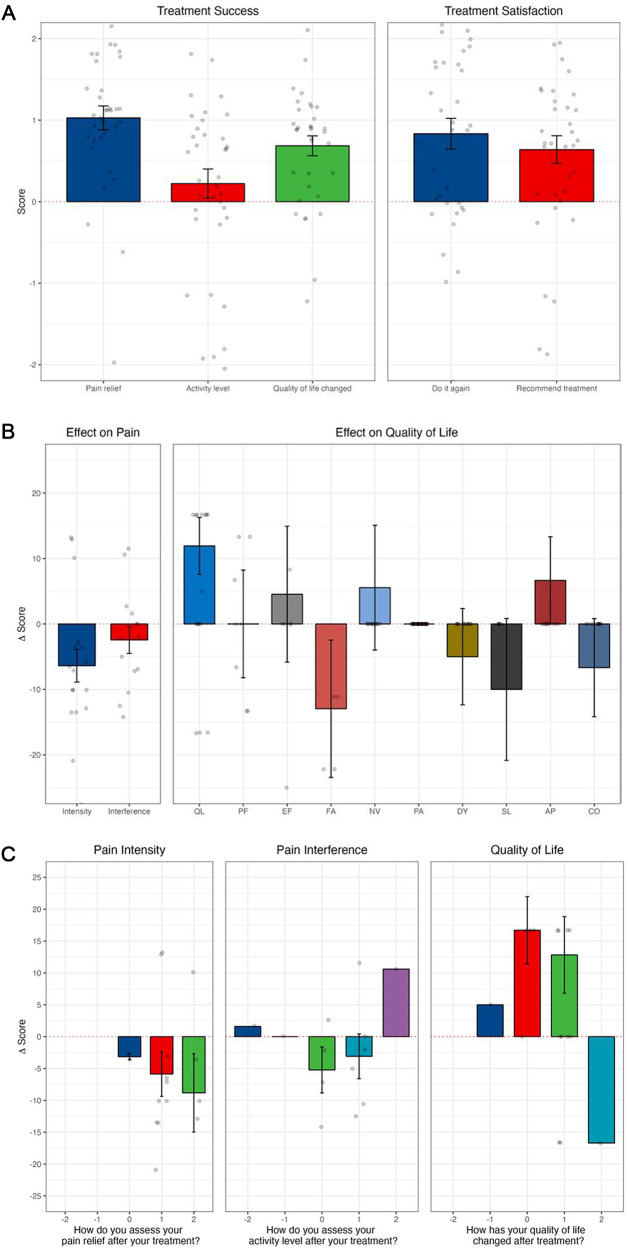
Outcomes following all interventional pain procedures: (A) treatment success and satisfaction by SAT; (B) PROMIS-pain intensity, PROMIS-pain interference, and EORTC QLQ-C15-PAL; (C) results from PROMIS and EORTC QLQ-C15-PAL correlating to subjective questions of significance from SAT. (A) The SAT is a 5-item simple question scale using a 5-point Likert scale, where the lower option (−2) indicated a negative response, the middle option (0) indicated a neutral response and the higher option (+2) positive response. (B) Effect on pain intensity and interference (PROMIS) and effect on subdomains of quality of life (EORTC QLQ-C15-PAL). EORTC QLQ-C15-PAL subdomains include AP appetite; CO constipation; DY dyspnea; EF emotional functioning; FA fatigue; NV nausea and vomiting; PA pain; PF physical functioning; QL quality of life; and SL sleeping difficulties. (C) Changes from baseline in pain intensity and pain interference from PROMIS, and changes in quality of life measured on EORTC QLQ-C15-PAL. Categories on the *x*-axis refer to SAT responses for pain relief, activity level, and quality of life as much worse (−2), somewhat worse (−1), no better and no worse (0), somewhat better (1), or much better (2). Change scores on the *y*-axis relate to changes in the overall quality of life score reported on PROMIS (pain intensity and pain interference) or EORTC QLQ-C15-PAL (quality of life).

There was a clinically and statistically significant reduction in adjusted (by age, sex, and baseline AKPS) morphine equivalent dose requirements (Table 3), pain intensity (PROMIS) and pain (EORTC-QLQ-C15-PAL), and global health status observed between day 1 and 14 post-procedure (Figure 1). There was an overall significant reduction in pain intensity (*p* < 0.01), a decrease in opioid consumption, and an improvement in quality of life (Table 4; Figure 1).

**Table 3.** Oral morphine equivalent dose and patient-reported outcome measures at baseline and 1 week after procedure

			**Baseline**			**One week post-procedure**		***p*-Values**	
Instruments		*N*	Mean (SD)	Median (IQR)	*N*	Mean (SD)	Median (IQR)	Univariate^†^	Multivariate^‡^
**OMED** (mg)		45	220.4 (208.4)	150.0 (95.0, 260)	41	157.6 (127.9)	127.0 (60.0, 210.0)	0.1	<0.01*
**PROMIS®**									
Pain Intensity		38	67.8 (8.0)	70.2 (61.9, 74.3)	20	64.8 (8.9)	64.9 (58.5, 72.0)	0.21	<0.01*
Pain Interference		36	69.2 (6.2)	69.2 (64.3, 76.3)	18	68.4 (5.3)	67.2 (65.0, 72.2)	0.69	0.32
**EORTC QLQ-C15-PAL**									
***Global health status***									
Global health status/QoL	(QL)	40	30.0 (23.6)	33.3 (16.7, 50.0)	21	38.3 (24.2)	38.3 (16.7, 66.7)	0.17	0.02*
***Functional scales***									
Physical functioning	(PF)	25	54.7 (22.0)	60.0 (46.7, 73.3)	15	55.6 (28.3)	60.0 (26.7, 83.3)	0.78	0.83
Emotional functioning	(EF)	29	33.1 (29.1)	41.7 (0.0, 50.0)	14	35.7 (19.7)	41.7 (23.0, 47.9)	0.93	0.73
***Symptom scales***									
Fatigue	(FA)	17	79.8 (18.9)	88.9 (66.7, 88.9)	14	83.4 (15.5)	88.9 (66.7, 97.2)	0.67	0.72
Nausea and vomiting	(NV)	34	42.2 (31.0)	33.4 (16.7, 50.0)	19	46.5 (32.2)	50.0 (16.7, 50.0)	0.60	0.71
Pain	(PA)	40	86.3 (18.8)	100.0 (66.7, 100.0)	21	72.2 (29.5)	83.3 (50.0, 100.0)	0.02*	<0.001*
Dyspnea	(DY)	39	23.9 (34.2)	0.0 (0.0, 33.3)	21	17.5 (22.7)	0.0 (0.0, 33.3)	0.51	0.17
Insomnia	(SL)	39	54.7 (35.5)	66.7 (33.3, 66.7)	21	49.2 (41.7)	66.7 (0.0, 100.0)	0.56	0.31
Appetite loss	(AL)	40	52.5 (36.9)	66.7 (25.0, 66.7)	21	58.7 (34.8)	66.7 (33.3, 100.0)	0.70	0.30
Constipation	(CO)	40	48.3 (35.4)	66.7 (25.0, 66.7)	21	42.9 (36.7)	33.3 (0.0, 66.7)	0.68	0.40

IQR = inter-quartile range; OMED = oral morphine equivalent dose; SD = standard deviation. PROMIS® scores were converted to T-scores as per the instrument scoring manual. EORTC QLQ-C15-PAL scores were transformed to 0–100 as per the EORTC QLQ scoring procedure;

*: statistical significance. *p*-Value from the ^†^ univariate linear model compared the averages of baseline to 1-week post-procedure and ^‡^multivariate linear mixed effect model adjusted for age, gender, and baseline AKPS scores, and accounted for within-patient variations.

**Table 4.** SAT item responses at 2 days and 1 week post-procedure

	***n***	Much worse	Somewhat worse	No better and no worse	Somewhat better	Much better	Mode
**Treatment success**							
*How do you assess your pain relief after the treatment?*	13	-	1 (8%)	2 (15%)	7 (54%)	3 (23%)	Somewhat better
*How do you assess your activity after the treatment?*	13	1 (8%)	3 (23%)	1 (8%)	8 (62%)	-	Somewhat better
*How has your quality of life changed after treatment?*	13	-	1 (8%)	6 (46%)	6 (46%)	-	No better and no worse and somewhat better
**Treatment satisfaction**							
	*n*	No definitely not	No, probably not	Unsure	Yes, probably	Yes, definitely	Mode
*Would you undergo this treatment again?*		-	1 (8%)	2 (15%)	5 (38%)	5 (38%)	Yes probably and Yes definitely
		Very much prefer my previous treatment	Somewhat prefer my previous treatments	No preference	Somewhat prefer this treatment to my previous treatment	Very much prefer this treatment to my previous treatment	Mode
*How do you compare the treatment you received to previous medication or therapies for you pain?*		-	-	5 (38%)	6 (46%)	2 (15%)	Somewhat prefer this treatment to my previous treatment

**Figure 2.**
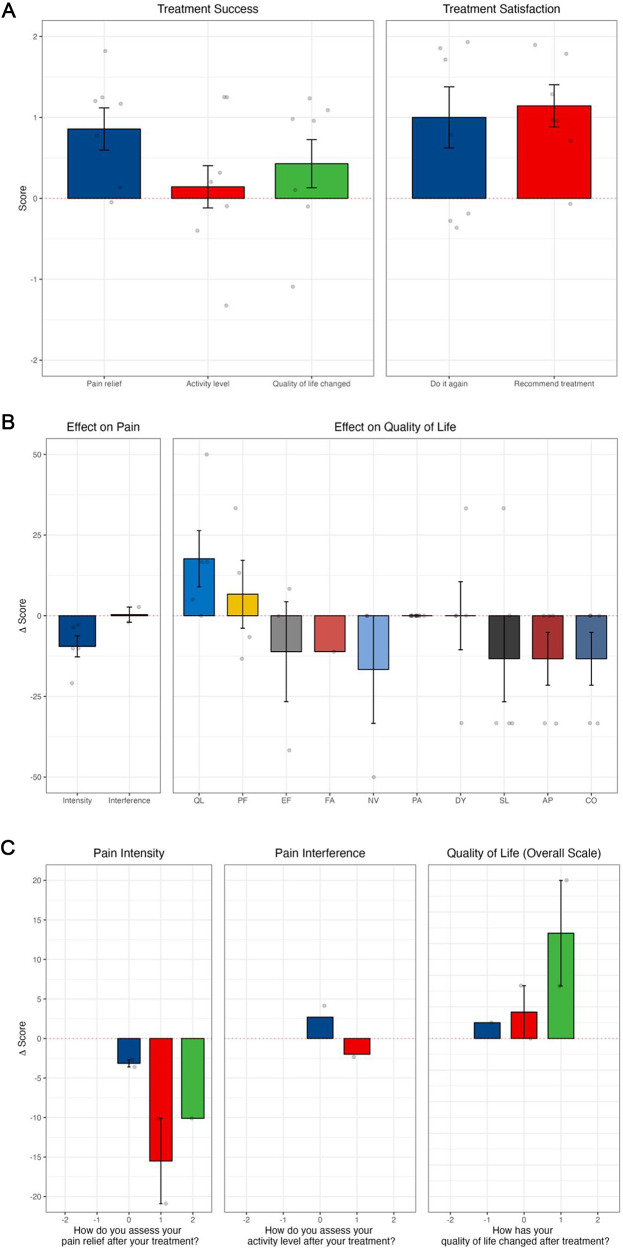
Outcomes following celiac plexus neurolysis (A) SAT; (B) PROMIS-pain intensity, PROMIS-pain interference, and EORTC QLQ-C15-PAL; and (C) results from PROMIS and EORTC QLQ-C15-PAL correlating to subjective questions of significance in SAT. Changes from baseline in pain intensity and pain interference from PROMIS, and changes in quality of life measured on EORTC QLQ-C15-PAL. Categories on the *x*-axis refer to SAT responses for pain relief, activity level, and quality of life as much worse (−2), somewhat worse (−1), no better and no worse (0), somewhat better (1), or much better (2). Change scores on the *y*-axis relate to changes in the overall quality of life score reported on PROMIS (pain intensity and pain interference) or EORTC QLQ-C15-PAL (quality of life).

**Figure 3.**
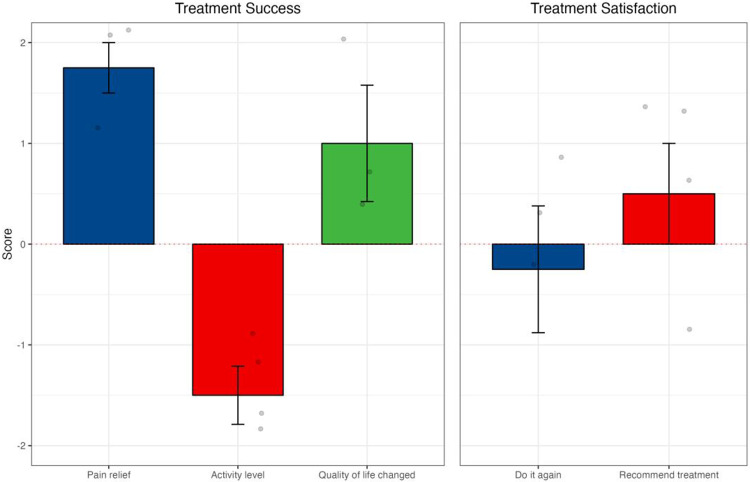
Outcomes following cordotomy: (A) SAT from cordotomy procedures; (B) PROMIS-pain intensity, PROMIS-pain interference, and EORTC QLQ-C15-PAL; and (C) results from PROMIS and EORTC QLQ-C15-PAL correlating to subjective questions of significance.

Similarly, when analyzing celiac plexus neurolysis in isolation, there were positive mean scores from SAT in all dimensions, and improvement of quality of life (Figure 2(A) and (B)). With cordotomy, despite a reduction in activity levels post-procedure, there was a positive change in pain relief and quality of life and reported treatment satisfaction (Figure 3).

In terms of adverse events, 3 patients who underwent celiac plexus neurolysis had transient fever, which improved within a few days. One patient developed bilateral lower limb weakness following bilateral thoracic cordotomy, which resolved spontaneously within 1 week.

### Mortality data

Twelve patients (25%) in the registry have since passed away. In this group, the mean survival time was 82.5 ± 70.62 days (range 25–233 days).

### Practicality of instruments included in the registry

The SAT showed reasonable acceptability (mean 1 week post-procedure pain relief was 1.1, suggesting “somewhat better”) with a completion rate of 50% at 1 week post-procedure (*n* = 24/48). However, less than half of the patients (*n* = 21/48; 43.7%) in the registry completed the post-procedure quality of life surveys (EORTC QLQ-C15-PAL).

## Discussion

This study was designed as a prototype to facilitate further development of a more widely applicable cancer pain intervention registry. It highlights the multidisciplinary approach to cancer pain management, including but not limited to general practitioners, oncologists, palliative medicine specialists, specialist pain medicine physicians, anesthetists, interventional radiologists, neurosurgeons, and gastroenterologists. Our study has used a pragmatic approach that serves both as a pilot implementation project for an interventional pain registry, while also collecting important clinical outcomes.

Patient outcomes following the interventional pain procedures were generally positive; however, a significant proportion of patients only benefited from it for a short period of time or for the remainder of their lives. Further work into optimizing the timing of the procedure, such as at the earlier stage of disease, may be considered. The registry only captured patients who had procedures undertaken, and not those patients who may have been appropriate for interventional pain procedures but were not referred for the service.

This registry captured a wide range of procedures, from peripheral nerve block to emerging treatments such as trans-arterial embolization. Previously, it has been difficult to access such integrated information through a single source or platform. The findings highlight the importance of collaborative care from the diverse specialties in managing cancer pain. Establishing the registry has served as a quality assurance process for the intervention practice as it has allowed direct and timely feedback to the clinical team, which will assist in improving future patient care. The registry has also shown signals of positive and negative clinical outcomes (clinical efficacy as measured by medication use, change of pain severity, and adverse events), which continues to generate additional information for systematic evaluation.

The most common interventional pain procedure reported in the registry was celiac plexus neurolysis, primarily for pancreatic cancer, with good evidence of support in the literature (Arcidiacono et al. 2011; Shwita et al. 2015). However, there was relatively limited information for different types of cancer, such as stomach cancer or cancer with metastasis into the liver and other upper gastrointestinal structures with this procedure. Moreover, there was a scarcity of data available on direct comparison for different approaches, including fluoroscopy, computed tomography, or endoscopic techniques. It is not clear whether this was due to a lack of availability or referral for these services, or because they were not considered appropriate in the individual patient context. Further details on the barriers and facilitators of interventional pain management for cancer pain from the perspective of clinicians were explored in a qualitative sub-study by Erciyas et al. (2024), which found that limited clinician knowledge may be one of the major barriers for patients accessing interventional pain management (Erciyas et al. 2024).

The relatively poor completion of some survey instruments in the registry highlights the need for more suitable instruments to maximize completion rate without compromising validity. For example, slightly greater proportions of patients (50%) completed the SAT, while quality of life measures were reported by only 43.7% of patients post-procedure. Others have reported the need to be flexible in the timing and method of data collection in this setting (Chatland et al. 2023), as questionnaire burden remains a barrier to completion of questionnaires by people undergoing largely palliative-based care (Dy et al. 2017). Not surprisingly, the completion rate in this study was comparable to that shown in a study specifically assessing patient-reported outcomes in palliative care units (Müller et al. 2023). In our study, the collection of data relied heavily on existing clinical staff, which may further explain the suboptimal collection of information. This also identifies the need for additional resources to facilitate implementation of the registry on a larger scale; or consideration to alternative methods (such as patient self-report via electronic means). In the future, survey instruments that take less time and effort for patients to complete, especially for those who are more advanced in their treatment journey, will be explored, such as the PEG, a 3-item scale to assess pain intensity (Krebs et al. 2009).

As is common in studies of advanced cancer, our study was limited by the amount of missing data in the post-procedure outcome measures. As described above, alternative measures for the collection of data, and the number of instruments and time-points included, should be considered. We also note there is heterogeneity in the population, and potentially outcomes should be stratified by predicted prognosis (e.g., patients with expected longer-term survival versus those with very limited prognoses) (Chow et al. 2008). Given the small number of centers involved, it is unclear how generalizable these findings are to the broader patient population in Australia, or indeed elsewhere. In particular, the challenges identified in these 2 adjacent centers may not fully reflect feasibility when extending to different sites. Additionally, the data on the range of procedures available, referral pathway, and clinical outcomes may be skewed due to differing resource and practice patterns. Obtaining more tailored information on them will be crucial before introducing it to new centers.

## Conclusion

This is a vital first step in creating a more widely applicable registry evaluating cancer pain interventions. It provided a systematic evaluation on the range of cancer pain intervention procedures available at a local level, as well as data on outcome measures using validated instruments. This allowed a timely review of clinical practice, which may lead to improvements in future patient care. Further expansion of the registry to other Australian hospitals is warranted.
